# Whole exome sequencing in Brugada and long QT syndromes revealed novel rare and potential pathogenic mutations related to the dysfunction of the cardiac sodium channel

**DOI:** 10.1186/s13023-022-02542-z

**Published:** 2022-10-27

**Authors:** Jia Chen, Hong Li, Sicheng Guo, Zhe Yang, Shaoping Sun, JunJie Zeng, Hongjuan Gou, Yechang Chen, Feng Wang, Yanping Lin, Kun Huang, Hong Yue, Yuting Ma, Yubi Lin

**Affiliations:** 1 The First Dongguan Affiliated Hospital, Guangdong Medical University, Dongguan, 523710 Guangdong Province China; 2 The Second Department of Cardiology, Department of Obstetrics and Gynecology, The Second People’s Hospital of Guangdong Province, Guangzhou, 510310 Guangdong Province China; 3 The University of Hong Kong-Shenzhen Hospital, Shenzhen, 518048 Guangdong Province China; 4 College of Plant Protection, Hunan Agricultural University, Changsha, 410128 Hunan Province China; 5 Department of Endocrinology and Metabolism, Zhuhai Hospital Affiliated to Jinan University, Zhuhai, 519000 Guangdong Province China; 6 Guangdong Provincial People’s Hospital, Guangdong Academy of Medical Sciences, Guangdong Geriatrics Institute, Guangdong Cardiovascular Institute, Guangzhou, 510080 Guangdong Province China; 7 College of Life Sciences, University of Chinese Academy of Sciences, Beijing, 100049 China

**Keywords:** Arrhythmia, Brugada syndrome, Long QT syndrome, Natriuretic peptide precursor A, Nebulette

## Abstract

**Background:**

Brugada syndrome (Brs) and long QT syndrome (LQTs) are the most observed “inherited primary arrhythmia syndromes” and “channelopathies”, which lead to sudden cardiac death.

**Methods:**

Detailed clinical information of Brs and LQTs patients was collected. Genomic DNA samples of peripheral blood were conducted for whole-exome sequencing on the Illumina HiSeq 2000 platform. Then, we performed bioinformatics analysis for 200 genes susceptible to arrhythmias and cardiomyopathies. Protein interaction and transcriptomic co-expression were analyzed using the online website and GTEx database.

**Results:**

All sixteen cases of Brs and six cases of LQTs were enrolled in the current study. Four Brs carried known pathogenic or likely pathogenic of single-point mutations, including *SCN5A* p.R661W, *SCN5A* p.R965C, and *KCNH2* p.R692Q. One Brs carried the heterozygous compound mutations of *DSG2* p.F531C and *SCN5A* p.A1374S. Two Brs carried the novel heterozygous truncated mutations (MAF < 0.001) of *NEBL* (p.R882X) and *NPPA* (p.R107X), respectively. Except for the indirect interaction between *NEBL* and *SCN5A*, *NPPA* directly interacts with *SCN5A*. These gene expressions had a specific and significant positive correlation in myocardial tissue, with high degrees of co-expression and synergy. Two Brs carried *MYH7* p.E1902Q and *MYH6* p.R1820Q, which were predicted as "damaging/possibly damaging" and "damaging/damaging" by Polyphen and SIFT algorithm. Two LQTs elicited the pathogenic single splicing mutation of *KCNQ1* (c.922-1G > C). Three LQTs carried a single pathogenic mutation of *SCN5A* p.R1880H, *KCNH2* p.D161N, and *KCNQ1* p.R243S, respectively. One patient of LQTs carried a frameshift mutation of *KCNH2* p. A188Gfs*143.

**Conclusions:**

The truncated mutations of *NEBL* (p.R882X) and *NPPA* (p.R107X) may induce Brugada syndrome by abnormally affecting cardiac sodium channel. *SCN5A* (p.R661W, p.R965C and p.A1374S) and *KCNH2* (p.R692Q) may cause Brugada syndrome*,* while *SCN5A* (p.R1880H)*, KCNQ1* (c.922-1G > C and p.R243S) and *KCNH2* (p.D161N and p.A188Gfs*143) may lead to long QT syndrome.

## Background

Inherited primary arrhythmia syndromes (IPAS), a rare disease (prevalence < 1/1,000) also called “channelopathies,” are commonly induced by genetic disorders and result in ventricular tachycardia (VT), torsade de pointe (TdP) and ventricular fibrillation (VF), consequently leading to sudden cardiac death (SCD) and even sudden unexplained death [1, 2]. The Brugada syndrome (Brs) and long QT syndrome (LQTs) are the most frequently observed IPAS in the general population. The prevalence of Brs and type-2/3 Brugada pattern electrocardiogram (ECG) is 0.5/1,000 and 6.1/1,000 and is reported to be the highest in Southeast Asia [3]. According to a study enrolling 44, 596 infants 15 to 25 days old (43, 080 whites) from 18 maternity hospitals, the prevalence of LQT between 451 and 470 ms of QTc might be close to 1:2000 [4]. A literature search reported that the prevalence of LQTs-induced sudden infant death syndrome (SIDS) ranged from approximately 3.9 to 20.6%, with an average of 12% [5]. The poor prognostic factors for mixed populations described in the series of Brs and LQTs, including sex (men for Brs, type-2 LQTs for female), symptoms, ECG characteristics, family history of SCD, genetic mutation, and inducibility of ventricular arrhythmia during the cardiac electrophysiological examination [6, 7].

For drug therapies of Brs, quinidine, blocking I_to_ and I_Kr_ channels reduces the arrhythmias incidence, including arrhythmic storms and multiple shocks, or as an alternative to an implantable defibrillator (ICD) in children at risk of arrhythmias. Additionally, isoproterenol, increasing the I_CaL_ inflow current, has been used successfully in cases of electrical storms [6]. Syncope in patients with LQTs is often triggered by periods of high sympathetic activity, including stress and exercise, for example, swimming. The type-1 LQTs patients should not be allowed to participate in competitive sports, especially swimming, or only cautiously with supervision. Type-2 and -3 LQTs patients are more susceptible to events during sleep. Type-2 LQTs patients are particularly sensitive to startle or sudden noises while sleeping, such as alarm clocks and telephones, and thus should avoid unexpected noises during sleep. ß blockers are recommended as the first line of therapy for all patients with LQTs. Patients with type-1 LQTs appear to benefit most from β blockers and should be started on β blockers as the first-line therapy [8]. According to the 2017 AHA/ACC/HRS guidelines, the ICD is the most important treatment for Brs and LQTs [9, 10]. The left cardiac sympathetic denervation should be considered for LQTs patients with β blockers therapy who continue to have syncope, TdP, recurrent appropriate ICD shocks despite antiarrhythmic drug therapy, or cardiac arrest [8].

Approximately 25 genes associated with Brs have been identified, of which eighteen genes are responsible for encoding ion channel subunits and seven genes for encoding regulatory proteins. Mutations on *SCN5A* are the most dominant for Brs and have more than 300 mutations related to Brs [11]. More than 20 disease-causing genes have been reported in almost 70% LQTs patients, including *KCNQ1* (30.1%, type-1 of LQTs), *KCNH2* (23.2%, type-2 of LQTs) and *SCN5A* (5.7%, type-3 of LQTs). However, the genetic causes for about one-third of LQTs remain unknown [12]. Notably, the genotype of *SCN5A* is a crucial component of the scheme for risk stratification of Brs and LQTs. It encodes Na_v_1.5, a sodium channel protein, wherein type-1 LQTs with mutations affecting the transmembrane domain or C-loop and type-2 and -3 LQTs with missense mutations on the S5-pore-S6 region have a considerably higher risk for cardiac events. Brs with pore-*SCN5A* mutation has a higher event risk than *SCN5A*-negative variants [1]. Based on these researches, the pathogenic genotypes of IPAS, for example, the Brs and LQTs are tightly associated with the risk of malignant cardiac events, especially ventricular arrhythmia and SCD. In this study, we enrolled twenty-two unrelated cases of Brs and LQTs. The potential pathogenic mutations carried by these patients will be identified by Whole Exome Sequencing (WES) to analyze the correlations among pathogenic mutations, clinical phenotypes and their risks. Interestingly, in these cases, we found that some common pathogenic genetic mutations may be related to Brs and LQTs. At the same time, we also first found and speculated that truncated *NEBL* and *NPPA* mutations might lead to Brs by aberrantly affecting the function of the cardiac sodium channels.

## Methods

### Study population and diagnostic criteria

Twenty-two cases of Brs and LQTs were enrolled from June 2015 to June 2017. Detailed clinical information was collected. The clinical information included family history, age of presentation, initial symptoms of VT, physical examination, ECGs, and monitoring of ICD based on their informed consent. The clinical diagnosis of Brs was based on the presence of typical type I Brugada pattern on the ECGs, characterized by a coved ST-segment and J-point elevation ≥ 0.2 mV in the right precordial leads [13]. The ECG's QTc (corrected for heart rate) can be calculated (QTc = QT interval + square root of the RR interval). The QTc interval helps us diagnose LQT. A QTc is prolonged if exceeding 0.47 s in women and 0.45 s in men [14, 15]. According to the Schwartz score, a definite LQTS is defined by an LQTS score ≥ 3.5 points [16].

### Ethics approval

This study was approved by the Guangdong Medical Institutional Review Board and Medical Ethics Committees [No. GDREC2016001H (R1)]. With the consent of the ethics committee, we followed up with the patients under the condition of informed consent and obtained blood samples for genetic analysis.

### Whole exome sequencing

Peripheral bloods from the patients were extracted for WES. Genomic DNA samples were isolated from peripheral blood using a standard DNA extraction protocol. The isolated genomic DNA was then fragmented into 150-200 bp and subjected to DNA library preparation using established Illumina paired-end protocols. Adaptor-ligated libraries were amplified via PCR. A portion of each library was used to create an equimolar pool. Each pool was amplified to enrich targets sequenced by the Agilent SureSelectXT Target Enrichment System (Agilent Technologies Inc., Santa Clara, CA, USA). According to the manufacturer's protocol, whole-exome capture was performed with the Agilent SureSelectXT Human All Exon 50 Mb Kit (Agilent Technologies Inc.). According to the manufacturer's instructions, the exome-enriched libraries were sequenced with the Illumina Hiseq 2000 platform (Illumina, San Diego, CA, USA), and 100 bp paired-end sequencing reads were generated. Each sample was sequenced per lane to obtain an average theoretical depth of 100 × [17, 18].

### Read mapping, variant detection, and functional annotation

After WES, raw reads were collected for quality control, in which low-quality reads were filtered, and 3′/5′ adapters were trimmed using the Trim Galore program (version 0.4.4). Clean reads were aligned to the human reference genome (University of California Santa Cruz, UCSC build hg19) using the Burrows-Wheeler Aligner (BWA, version: 0.7.17-r1188) program. The quality scores were recalibrated, and reads were realigned to the reference genome using the Genome Analysis Toolkit (GATK, version: 3.5-0-g36282e4) software package. Following the exclusion of duplicate reads, insertion-deletions (InDels) and single-nucleotide polymorphisms (SNPs) were called using the GATK or Sequence Alignment/Map tools (SAM tools, Version: 1.3.1). The quality value of variants detected by GATK was 99 (the highest value), and the variant abundance was more than 30% [17, 18].

### Pathogenic risk classification

The SNPs and Indels were annotated using a pipeline, in which all insertion and deletion variants occurring at coding regions were considered damaging, and nonsynonymous SNPs were predicted by SIFT (http://sift.jcvi.org/www/) and PolyPhen-2 (Polymorphism Phenotyping v2, http://genetics.bwh.harvard.edu/pph2/) [19]. Subsequently, the common risk genes associated with cardiomyopathies and arrhythmias, as reported in our previous research [18, 20], were detected in the patients. These variants were screened with the following filtering criteria: (1) same variants in the WES data; (2) missense, nonsense, insertion and deletion variants; (3) SNPs with minor allele frequency, not ≥ 0.01 according to the SNP database of National Center; excluded variants with allele frequency in 1000genomes (2015 version) higher than 1%, or higher than 5% in house frequency. The potential risk variants were classified as “pathogenic (P)”, “likely pathogenic (LP)”, “uncertain significance (VUS)”, “likely benign (LB)” or “benign (B)” by the Clinvar database [17, 18] and InterVar tool [21] following the 2015 ACMG/ACP guidelines [22]. The detailed ACMG classification was shown in our previous research [18].

### Protein interaction analysis

Using the online website https://string-db.org/, the target gene was input for protein–protein interaction analysis. The combined score between proteins with interaction records was scored by combining other database records, experimental verification, gene fusion, co-localization, co-expression and homology analysis. It is currently the mainstream and high-reliability database of protein-interaction information.

### Transcriptomic co-expression analysis

In the Genotype-Tissue Expression (GTEx) database [23], the TPM matrix of ventricular tissue, spleen, whole blood, ovary, lung and liver were used for co-expression analysis. The GTEx version was GTEx analysis V8 (dbgap access phs000424.v8.p2). The "Cor" function in the R language was used to calculate the gene correlation matrix. The method parameter used Spearman correlation, in which the correlation threshold was above 0.7, indicating a very close relationship; 0.4–0.7 indicated a close relationship; 0.2–0.4 indicated a general relationship.

## Results

### ***Genotype***–***phenotype relationship***

In all, sixteen cases of Brs (median onset-age, 46-year-old; IQR 21.5-year-old; 22 to 65-year-old) and six cases of LQTs (median onset-age, 15-year-old; IQR 18-year-old; 6 to 55-year-old) were enrolled in the current study (Table 1). The echocardiograms (ECGs) of these patients showed normal cardiac structure. VT or VF was detected in 19 cases. Two cases of Brs were induced VF by electrophysiological examination (EPS). These patients suffered from clinical symptoms, including dizziness, syncope, palpitation, amaurosis, and chest distress. Nineteen cases were implanted with ICD, while three Brs refused ICD implantation. Two cases of Brs and one case of LQTs had a familial history of SCD. One case of Brs was the dominant familial inheritance because three siblings had Brugada-like ECGs without clinical symptoms.

**Table 1:** The clinical characteristics of patients with Brugada syndrome and long QT syndrome

No	DS	Sex	Age (years)	Onset of age (years)	Ventricular arrhythmia	Symptoms	ICD therapy	Drugs	Familial history
1	Brs	F	40	38	VT	Dizzy, syncope	refused	Beta blocker	No
2	LQTs	F	61	55	VF	Syncope	ICD	Beta blocker	No
3	Brs	M	72	65	VT	Syncope	refused	Beta blocker	Brother (SCD, 31-year-old)
4	Brs	M	48	47	VF	Amaurosis, syncope	ICD	No	No
5	Brs	M	46	45	VT, VF	Dizzy, amaurosis, syncope	ICD	No	No
6	Brs	M	60	57	VT, VF	Syncope	ICD	Beta blocker, mexiletine	No
7	Brs	M	57	47	No	Syncope	refused	No	Three brothers (Brs)
8	Brs	M	41	13	VF	Syncope	ICD	No	No
9	Brs	M	49	47	VT	Palpitation, chest distress	ICD	No	No
10	Brs	M	63	53	EPS induced VF	Dizzy, amaurosis,	ICD	No	No
11	Brs	M	22	22	VF	Syncope	ICD	No	No
12	Brs	F	51	51	VF	Syncope when wake up	ICD	No	No
13	LQTs	F	13	6	Tdp, VF	Sleeping syncope	ICD	Mexiletine	No
14	LQTs	F	19	16	VF	Palpitation, amaurosis, Syncope	ICD	Beta blocker, potassium magnesium aspartate	No
15	Brs	M	41	34	VF	Syncope	ICD	Beta blocker	No
16	Brs	M	54	53	EPS induced VF	Amaurosis, palpitation	ICD	No	No
17	Brs	M	32	31	VT, VF	Syncope, convulsion	ICD	Beta blocker, potassium	No
18	Brs	M	33	23	No	No	ICD	No	No
19	LQTs	F	35	30	VF	Syncope	ICD	Beta blocker	No
20	Brs	M	41	30	VF, AF	Amaurosis, syncope	ICD	No	No
21	LQTs	F	16	14	VF	Amaurosis, syncope	ICD	Beta blocker, pacing rate of 95 bpm	No
22	LQTs	F	16	12	VF	Syncope, chest distress	ICD	No	Mother (SCD, 33-year-old)

*DS* diseases, *M* male, *F* female, *AF* atrial fibrillation, *VT* ventricular tachycardia, *VF* ventricular fibrillation, *Tdp* torsades de pointes, *EPS* electrophysiology study, *SCD* sudden cardiac death, *ICD* Implantable Cardioverter-Defibrillator,- loss of follow-up or lack of clinical data due to refuse of hospitalization

The WES detected some known and pathogenic/likely-pathogenic (P/LP) mutations. Four cases of Brs demonstrated single mutations with known or likely pathogenicity, including p.A1374S (Clinic/ACMG = LP/VUS, No.5, VT/VF, ICD therapy), p.R661W (Clinic/ACMG = P/VUS, No.8, VF, ICD therapy), and p.R965C (Clinic/ACMG = LP/VUS, No.10, VF induced by EPS, ICD therapy) on *SCN5A*, and p.R692Q (Clinic/ACMG = LP/VUS, No.18, ICD therapy) on *KCNH2*. One case of Brs carried the compound heterozygous and pathogenic mutations of *DSG2* p.F531C (Clinic/ACMG = LP/LP) and *SCN5A* p.A1374S (Clinic/ACMG = LP/VUS, No.11, VF, ICD therapy). Two cases of LQTs elicited the pathogenic and single splicing mutation of *KCNQ1* c.922-1G > C (Clinic/ACMG = P/P, No.19, VF, ICD therapy). Three cases of LQTs carried a single pathogenic mutation of *SCN5A* p.R1880H (Clinic/ACMG = P/VUS, No.13, TdP and VF, ICD therapy), *KCNH2* p.D161N (Clinic/ACMG = P/LP, No.21, VF, ICD therapy), and *KCNQ1* p.R243S (Clinic/ACMG = P/LP, No.22, familial history of SCD, VF, ICD therapy), respectively (Table 2).

**Table 2:** The known and likely pathogenic mutations of Brugada syndrome and long QT syndrome

ID	DS	Chr	Start	Gene	Amino acid change	Het	1000 g/Esp	SIFT	Polyphen	Clinic	ACMG	Evidence	dbSNP
2	LQTs	chr11	2,604,664	*KCNQ1*	NM_000218:exon7:c.922-1G > C	±	–	–	–	P	P	PVS1, PM2_Supporting, PP4, PP1	rs387906290
5	Brs	chr3	38,598,739	*SCN5A*	NM_001160161:exon23:c.G4120T:p.A1374S	±	0.001	0.00(D)	1.00(D)	LP	VUS	PM2_Supporting, PM1, PP3	rs200034939
8	Brs	chr3	38,640,451	*SCN5A*	NM_000335:exon13:c.C1981T:p.R661W	±	< 0.001	0.00(D)	1.00(D)	P	VUS	PM2_Supporting, PP3	rs199473139
10	Brs	chr3	38,622,757	*SCN5A*	NM_000335:exon17:c.C2893T:p.R965C	±	0.001	0.00(D)	1.00(D)	LP	VUS	PM2_Supporting, PS4_M, PS3_Supporting, PP3	rs199473180
11	Brs	chr18	29,116,333	*DSG2*	NM_001943:exon11:c.T1592G:p.F531C	±	–	0.00(D)	1.00(D)	LP	LP	PM2_Supporting, PM3_Strong, PS3_Supporting	rs200484060
		chr3	38,598,739	*SCN5A*	NM_001160161:exon23:c.G4120T:p.A1374S	±	0.001	0.00(D)	1.00(D)	LP	VUS	PM2_Supporting, PS4_Supporting, PP3	rs200034939
13	LQTs	chr3	38,592,170	*SCN5A*	NM_001099405:exon27:c.G5639A:p.R1880H	±	< 0.001	0.06(T)	0.99(D)	P	VUS	PM2_Supporting, PS4_Supporting, PP3	rs370694515
18	Brs	chr7	150,644,473	*KCNH2*	NM_172057:exon9:c.G2075A:p.R692Q	±	0.001	0.58(T)	1.00(D)	LP	VUS	PM2_Supporting, PP2	rs199473020
19	LQTs	chr11	2,604,664	*KCNQ1*	NM_000218:exon7:c.922-1G > C	±	–	–	–	P	P	PVS1, PM2_Supporting, PP4, PP1	rs387906290
21	LQTs	chr7	150,649,569	*KCNH2*	NM_001204798:exon2:c.G481A:p.D161N	±	–	0.00(D)	1.00(D)	P	LP	PM2_Supporting, PS4_M, PS3_Supporting, PP2, PP3	rs199472912
22	LQTs	chr11	2,593,286	*KCNQ1*	NM_000218:exon5:c.C727A:p.R243S	±	–	0.00(D)	1.00(D)	P	LP	PM2_Supporting, PM5, PM1, PP3	rs199472713

*DS* diseases, *LQTs* long QT syndrome, *Brs* Brugada syndrome, *Chr* chromosome, *1000G/Esp* 1000genomes (2015 version) or Esp6500 database, *SNP* single nucleotide polymorphism, *PP* polyphen-2, *D* damaging, *B* benign, *T* tolerated, ± heterozygous carrier, *P* pathogenic, *LP* likely pathogenic, – no report

**Fig. 1:**
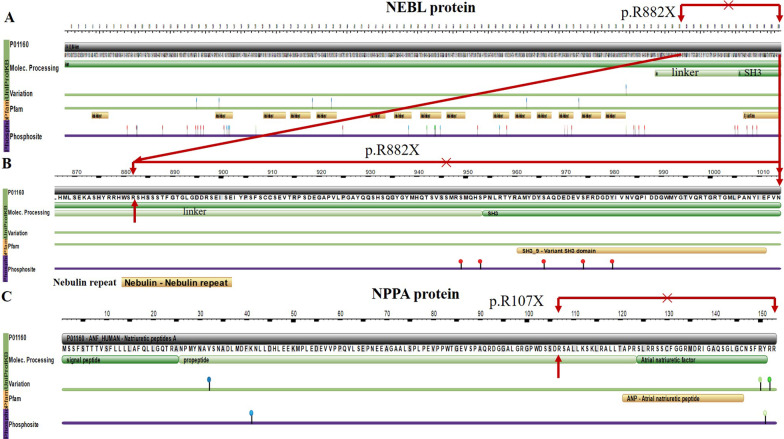
The changes in amino acids of NEBL and NPPA proteins induced by the truncated mutations

We also found several novel mutations potentially associated with Brs and LQTs. In two Brs patients, we first found the heterozygous p.R882X (Clinic/ACMG = VUS/VUS, No.1, VT, refused ICD therapy) of the *NEBL* gene (at the rs151012132 locus) and p.R107X (Clinic/ACMG = -/LP, No.3, VT, family history of SCD, refused ICD therapy) of the *NPPA* gene (Table 3), respectively, as truncating mutations, which were absent from or found with MAF (minor allele frequency) < 0.001 in the 1000genomes population. *NEBL* p.R882X may induce the loss of domains of partial linker and SH3 in NEBL protein (Fig. 1A, B). *NPPA* p.R107X only expressed the pro-peptide (Fig. 1C) but lost the effective structure of atrial natriuretic peptide (ANP).

**Table 3:** The risk mutations of Brugada syndrome and long QT syndrome

ID	DS	Chr	Start	Gene	Amino acid change	Het	1000 g/Esp	SIFT	Polyphen	Clinic	ACMG	Evidence	dbSNP
1	Brs	chr1	3,342,629	*PRDM16*	PNM_199454:exon14:c.G3124A:p.G1042R	±	–	0.00(D)	1.00(D)	–	VUS	PM2_Supporting	–
		chr10	21,097,556	*NEBL*	NM_006393:exon26:c.C2644T:p.R882X	±	< 0.001	–	–	VUS	VUS	PM2_Supporting	rs151012132
		chr2	179,447,747	*TTN*	NM_003319:exon141:c.G38588A:p.R12863Q	±	–	0.04(D)	1.00(D)	VUS	VUS	PM2_Supporting	–
		chr2	179,460,249	*TTN*	NM_003319:exon123:c.A30637G:p.I10213V	±	–	0.36(T)	0.95(D)	–	VUS	PM2_Supporting	rs56025724
		chr21	18,919,405	*CXADR*	NM_001207063:exon2:c.A104G:p.E35G	±	–	0.05(T)	1.00(D)	–	VUS	PM2_Supporting	–
3	Brs	chr1	11,907,301	*NPPA*	NM_006172:exon2:c.C319T:p.R107X	±	–	–	–	–	LP	PM2_Supporting， PVS1	–
		chr1	228,467,100	*OBSCN*	NM_001098623:exon27:c.T7351G:p.F2451V	±	–	0.27(T)	0.93(D)	–	VUS	PM2_Supporting	–
		chr1	228,547,344	*OBSCN*	NM_052843:exon81:c.C18751T:p.R6251W	±	–	0.02(D)	0.74(P)	–	VUS	PM2_Supporting	–
		chr1	228,559,174	*OBSCN*	NM_001098623:exon94:c.C20695T:p.R6899W	±	–	0.00(D)	0.64(P)	–	VUS	PM2_Supporting	–
		chr2	179,640,347	*TTN*	NM_003319:exon27:c.G6106A:p.E2036K	±	–	0.27(T)	1.00(D)	–	VUS	PM2_Supporting	–
4	Brs	chr12	114,793,662	*TBX5*	NM_080717:exon8:c.C1082T:p.T361I	±	< 0.001	0.13(T)	0.46(P)	–	VUS	PM2_Supporting	rs267603320
		chr2	179,432,053	*TTN*	NM_003319:exon154:c.C51611T:p.S17204F	±	–	0.00(D)	0.84(P)	–	VUS	PM2_Supporting	–
6	Brs	chr15	39,885,760	*THBS1*	NM_003246:exon19:c.C3158T:p.T1053M	±	< 0.001	0.02(D)	0.71(P)	–	VUS	PM2_Supporting	rs267604168
		chr17	39,915,014	*JUP*	NM_002230:exon9:c.C1606G:p.Q536E	±	–	0.03(D)	0.30(B)	–	VUS	PM2_Supporting	–
		chr3	71,015,109	*FOXP1*	NM_001244813:exon14:c.C1521G:p.N507K	±	–	0.04(D)	0.83(P)	–	VUS	PM2_Supporting	–
7	Brs	chr19	16,593,346	*CALR3*	NM_145046:exon7:c.G833A:p.R278H	±	–	0.03(D)	0.00(B)	–	VUS	PM2_Supporting	–
		chr21	18,937,961	*CXADR*	NM_001338:exon7:c.C1049T:p.A350V	±	–	0.102(T)	0.949(D)	–	VUS	PM2_Supporting	–
		chr4	111,539,442	*PITX2*	NM_000325:exon3:c.G814A:p.A272T	±	–	0.41(T)	0.95(D)	–	VUS	PM2_Supporting	–
		chr5	251,519	*SDHA*	NM_001294332:exon12:c.A1586C:p.Q529P	±	–	0.02(D)	0.99(D)	–	VUS	PM2_Supporting	–
9	Brs	chr5	37,333,576	*NUP155*	NM_001278312:exon13:c.C1507T:p.L503F	±	–	0.01(D)	1.00(D)	–	VUS	PM2_Supporting	–
		chr7	128,481,334	*FLNC*	NM_001127487:exon12:c.G1924A:p.V642I	±	< 0.001	0.82(T)	0.67(P)	–	VUS	PM2_Supporting	rs369387744
12	Brs	chr10	112,581,622	*RBM20*	NM_001134363:exon11:c.T3245G:p.L1082R	±	–	0.00(D)	0.08(B)	–	VUS	PM2_Supporting	–
		chr20	33,345,504	*NCOA6*	NM_001242539:exon7:c.G1047C:p.L349F	±	–	0.02(D)	0.89(P)	–	VUS	PM2_Supporting	–
14	LQTs	chr1	228,467,732	*OBSCN*	NM_001098623:exon28:c.G7607C:p.G2536A	±	–	0.01(D)	1.00(D)	–	VUS	PM2_Supporting	–
		chr3	38,739,348	*SCN10A*	NM_001293307:exon26:c.T5069C:p.M1690T	±	–	0.00(D)	0.99(D)	–	VUS	PM2_Supporting	–
		chr3	38,770,058	*SCN10A*	NM_001293307:exon14:c.C2321T:p.T774M	±	–	0.93(T)	0.02(B)	–	VUS	PM2_Supporting	–
		chr7	150,655,499	*KCNH2*	NM_000238:exon4:c.563_564del:p.A188Gfs*143	±	–	–	–	–	LP	PM2_Supporting, PVS1	–
15	Brs	chr14	23,853,757	*MYH6*	NM_002471:exon36:c.G5459A:p.R1820Q	±	< 0.001	0.01(D)	1.00(D)	–	VUS	PM2_Supporting	rs371222772
		chr19	39,406,284	*SARS2*	NM_017827:exon16:c.C1519T:p.R507W	±	< 0.001	0.01(D)	0.54(P)	–	VUS	PM2_Supporting	rs143316017
		chr6	152,472,791	*SYNE1*	NM_033071:exon134:c.C24134T:p.A8045V	±	–	0.12(T)	0.98(D)	–	VUS	PM2_Supporting	–
16	Brs	chr14	74,970,636	*LTBP2*	NM_000428:exon31:c.4573_4575del:p.1525_1525del	±	–	–	–	–	VUS	PM2_Supporting, PM4_Supporting	–
		chr2	179,453,729	*TTN*	NM_003319:exon132:c.G35528A:p.R11843Q	±	0.001	0.10(T)	1.00(D)	–	VUS	PM2_Supporting	rs377203669
		chr2	179,455,524	*TTN*	NM_003319:exon132:c.C33733T:p.R11245C	±	0.001	0.00(D)	1.00(D)	–	VUS	PM2_Supporting	rs200898955
		chr7	140,624,425	*BRAF*	NM_004333:exon1:c.G79A:p.A27T	±	–	0.57(T)	0.48(P)	–	VUS	PM2_Supporting	–
		chr8	106,573,686	*ZFPM2*	NM_012082:exon4:c.A397G:p.M133V	±	–	0.60(T)	0.01(B)	–	VUS	PM2_Supporting	rs77117583
17	Brs	chr1	228,547,680	*OBSCN*	NM_052843: exon81:c.G19087A:p.G6363S	±		1.00(T)	0.02(B)	–	VUS	PM2_Supporting	–
		chr14	23,883,054	*MYH7*	NM_000257: exon39:c.G5704C:p.E1902Q	±	< 0.001	0.08(D)	0.61(P)	–	VUS	PM2_Supporting	rs187073962
20	Brs	chr6	112,506,509	*LAMA4*	NM_001105206:exon9:c.A1007G:p.K336R	±	–	0.57(T)	0.52(P)	–	VUS	PM2_Supporting	–

*DS* diseases, *LQTs* long QT syndrome, *Brs* Brugada syndrome, *Chr* chromosome, *1000G/Esp* 1000genomes (2015 version) or Esp6500 database, *SNP* single nucleotide polymorphism, *PP* polyphen-2, *D* damaging, *B* benign, *T* tolerated, ± heterozygous carrier, *P* pathogenic, *LP* likely pathogenic, – no report

The variants of *MYH7* (p.E1902Q, rs187073962, Clinic/ACMG = -/VUS, No.17, VT/VF, ICD therapy) and *MYH6* (p.R1820Q, rs371222772, Clinic/ACMG = -/VUS, No.15, VF, ICD therapy), predicted as “damaging/possibly damaging” and “damaging/damaging” by Polyphen and SIFT algorithms, were demonstrated in patients of Brs. A patient of LQTs carried a frameshift mutation of *KCNH2* p.A188Gfs*143 (Clinic/ACMG = -/LP, No.13, TdP and VF, ICD therapy), which did not exist in the 1000genomes population.

### Literature summary of NEBL and SCN5A interaction

**Fig. 2:**
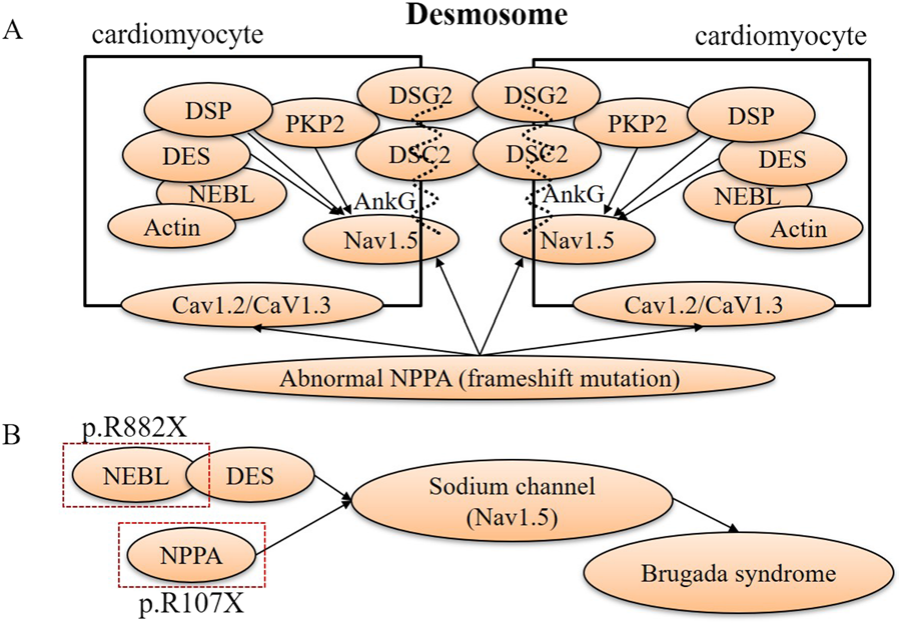
The interactions among *NEBL*, *NPPA* and *SCN5A* associated with Brugada syndrome. The desmosome proteins of cardiomyocytes include desmoglein-2 (DSG2), desmocollin-2 (DSC2), plakophilin-2 (PKP2), desmoplakin (DSP), desmin (DES). *SCN5A* encoded Na_v_1.5 protein, as a subunit of the cardiac sodium channel. Ankyrin-G (AnkG) promotes the Na_v_1.5 anchoring and localizing to the cell membrane. Cav1.2 and Cav1.3 are the subunits of the L-type calcium channel. According to previous research, the arrows illustrate that DSG2, PKP2, DSP, and DES dysfunction would abnormally regulate sodium channel function (Na_v_1.5). NEBL, nebulin-like protein. NPPA, natriuretic peptide precursor A

According to previous studies, abnormal desmosome genetic expressions, including desmocollin-2 (*DSC2*), desmoglein-2 (*DSG2*), plakophilin-2 (*PKP2*), desmoplakin (*DSP*), plakoglobin (*JUP*) and desmin (*DES*) participate in the pathogenic mechanism of arrhythmogenic cardiomyopathy (ACM) [17, 18, 20, 24–27]. Interestingly, loss-of-function of *SCN5A* mutations induced complex arrhythmia, including Brs, atrial fibrillation (AF), atrial standstill, VT and sick sinus syndrome [28]. In this study, we first discovered some interesting interactions among desmosome proteins and cardiac sodium channels in cardiomyocytes, including *DSG2* and Na_v_1.5 (α subunit of the sodium channel, encoded by *SCN5A*), *PKP2* and Na_v_1.5, *DES* and Na_v_1.5, *NEBL* and *DES* in the cardiac desmosomes, through literature research using “*NEBL* and *SCN5A* (or Na_v_1.5, or sodium channel), nebulette and *SCN5A* (or Na_v_1.5, or sodium channel), each protein of desmosomes (including *DSG2*, *DSC2*, *PKP2* and *DSP*) and *NEBL* (or nebulette), each protein of desmosomes (including *DSG2*, *DSC2*, *PKP2* and *DSP*) and *SCN5A* (or Na_v_1.5, or sodium channel), *NEBL* (or nebulette) and Brugada syndrome, each protein of desmosomes (including *DSG2*, *DSC2*, *PKP2* and *DSP*) and Brugada syndrome” in the NCBI PubMed database. We summarized these literatures related to *NEBL*, desmosome proteins and Na_v_1.5 as follows (shown in Fig. 2A, B**)**.

*NEBL* encodes a nebulin-like protein expressed in cardiac muscle. This protein binds to actin, interacting with thin filaments and Z-line-associated proteins in striated muscle and cardiac myofibril assembly. NEBL plays a vital role in the dynamics of the DES-NEBL-actin complex in cardiac myocytes and maintains the relaxation–contraction cycles of the heart. The NEBL exhibits high-affinity interaction and synergic action with DES filaments and is a direct linker between actin and DES. The pathogenic mutations of *NEBL* will induce dilated cardiomyopathy, hypertrophic cardiomyopathy, left ventricular non-compaction cardiomyopathy, and endocardial fibroelastosis [24–27].

Additionally, the pathogenic mutants E245D, T453I, and knockout of *DES* increase binding affinity for NEBL, delay filament assembly kinetics, and cause significant attenuation and disruption of cardiac actin-NEBL- DES-Z lines filament network as dynamic DES assembly [29]. The pathogenic mutations of *DES* can cause severe impairment of filament formation and induce ACM, consequently complicating rhythm disorder, conduction disease, and heart failure [30]. Therefore, the underlying mechanism of NEBL-inducing cardiomyopathies may be comparable to DES. According to previous studies, some cases of ACM overlap the phenotype of Brs [31, 32]. Like DES, PKP2 is one of the critical components in desmosomes of the intercalated disk. It is necessary to maintain gap junction integrity and formation through the DES-DSP-PKP2 complex in desmosomes. The lost expression of PKP2 decreases and disrupts the expression and trafficking of the sodium channel (Na_v_1.5) at the intercalated disc, which can degrade cardiac sodium current and subsequently lead to overlapped phenotypes of ACM and Brs [33–36]. Based on these evidences, we proposed that the mutation of *NEBL* might theoretically associate with Brs through the interaction of abnormal NEBL protein with the sodium channel, which has not been demonstrated yet.

### Literature summary of NPPA and SCN5A interaction

We also first discovered obvious interactions between natriuretic peptide precursor A (*NPPA*) and Na_v_1.5 through current summating research from the NCBI PubMed database using “*NPPA and SCN5A* (or Na_v_1.5, or sodium channel), natriuretic peptide precursor A and *SCN5A* (or Na_v_1.5, or sodium channel), ANP and *SCN5A* (or Na_v_1.5, or sodium channel), Brugada syndrome and *NPPA* (or ANP, or natriuretic peptide precursor A)”. The literature summary related to *NPPA* and Na_v_1.5 was as follows (shown in Fig. 2A, B).

*NPPA* encodes ANP, expressed in the embryo's atrial and ventricular myocardium. *NPPA* is also expressed in the adult heart but is downregulated in the ventricles around birth to become restricted to the atria and the ventricular conduction system. In a previous study, for atrial myocyte of transgenic mice carrying a frameshift mutation of *NPPA*, the expression, currents (I_Na_ and I_CaL_) and action potential duration of cardiac sodium (Na_v_1.5) and L type calcium (Ca_v_1.2/Ca_v_1.3) channels were significantly reduced. In contrast, the rectifier potassium channel current (I_Ks_) markedly increased compared to the wild type of *NPPA*. The malignant changes induced by the frameshift *NPPA* mutation create an atrial substrate of recurrent AF. It is worth noting that ANP is expressed in the atrium and the ventricle. Especially, ANP expression is more significantly re-induced in the ventricles in response to pathological cardiac stress, such as cardiac hypertrophy or myocardial infarction [37]. There were also obvious interactions among *NPPA*, Na_v_1.5 and CaV1.2/CaV1.3 (I_CaL_). The truncated NPPA may induce Brs through the impact on the function of the sodium channel.

### NEBL and NPPA interact and co-expressed with SCN5A

**Fig. 3:**
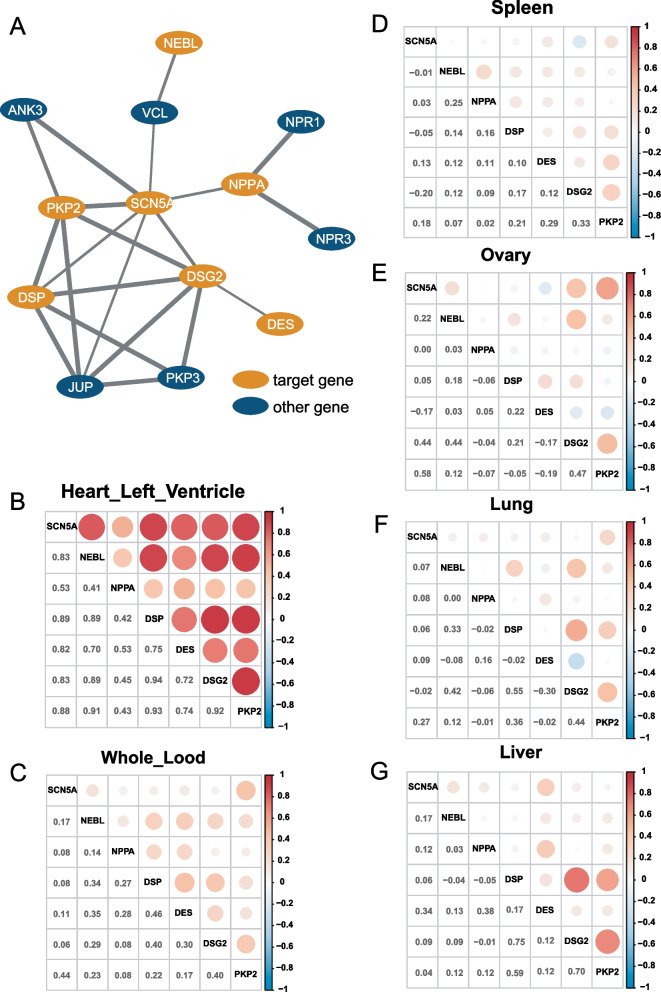
Protein interaction and transcriptomic co-expression analysis

The genes of cardiac desmosome components include *DSG2*, *DSC2*, *PKP2*, *DSP*, *JUP* and *DES*. We analyzed the protein interactions corresponding to these genes to test our hypothesis. In the existing interaction database, PPI interaction network analysis shows that these genes have significant interaction (Fig. 3A). The genes including *DSG2*, *PKP2*, *DSP* and *JUP* directly interact with *SCN5A*. There is indirect interaction between *NEBL/DES* and *SCN5A*, while *DES* has indirect interaction with *SCN5A* through *DSG2*. VCL connects the indirect interaction between NEBL and SCN5A. Vinculin protein encoded by *VCL* is a cytoskeleton protein related to extracellular matrix adhesion and connection, and its mutation may lead to dilated and hypertrophic cardiomyopathy.

In addition, we downloaded the expression data from six different tissue sources (including ventricular tissue, whole blood, spleen, ovary, lung and liver) from the public database of GTEx, and calculated the correlation of the expression of these genes (including *SCN5A*, *NEBL*, *NPPA*, *DSP*, *DES*, *DSG2* and *PKP2*) in each tissue (Fig. 3B–G). It was found that the expression of these genes had the highest correlation in cardiac tissue. The correlation between *SCN5A* and *NEBL* reached 0.83, and the correlation between *SCN5A* and *NPPA* also reached 0.53. The correlations between SCN5A and other genes (including *DSP*, *DES*, *DSG2* and *PKP2*) are significantly positive between 0.41 and 0.94, with high degrees of co-expression and synergy. In other tissues, these genes' co-expression has low or no correlation. Therefore, we verified significant co-expression and protein interaction between *NPPA*, *NEBL*, *SCN5A*, *DSP*, *DES*, *DSG2* and *PKP2* genes.

## Discussion

Our study enrolled twenty-two cases of Brs and LQTs and conducted WES for these cases to explore the potential pathogenic mutations. Interestingly, according to genotype-phenotype, protein interaction and transcriptomic co-expression analysis, we first found that the truncated mutations of *NEBL* and *NPPA* might induce Brs through the abnormal impact on the function of the cardiac sodium channel. Additionally, *SCN5A* (p.R661W, p.R965C and p.A1374S) and *KCNH2* (p.R692Q) may cause Brs, while *SCN5A* (p.R1880H), *KCNQ1* (c.922-1G > C and p.R243S) and *KCNH2* (p.D161N and p.A188Gfs*143) may lead to LQTs.

### *NEBL* and *NPPA* mutations may induce Brugada syndrome by aberrantly affecting the cardiac sodium channel

The cardiac actin-NEBL-DES-Z lines filament network participates in the maintenance of the desmosome junction and the stability of the myocardial structure. As reported before, *NEBL* p.G202R can augment desmosome separation. The *NEBL* p.A592E presents abnormal ultrastructure changes and DES downregulation [38]. A GWAS analysis has revealed that *NEBL* p.A219D (rs2296610) is significantly correlated with AF [39], suggesting that the *NEBL* mutation may probably associate with an increased risk of arrhythmia. *NPPA* mutation has been disclosed to link with familial AF, increasing the risk of AF [40] and stroke (*NPPA* p.V32M) [41]. The heterozygous mutation of *NPPA* p.S64R caused refractory AF due to the augmented potassium current and shortened atrial action potential [42, 43]. The homozygous mutation of *NPPA* p.R150Q is associated with dilated cardiomyopathy with atrial standstill [44]. *NPPA* p.I138T causes AF by activating TNF-α, NF-κB, and IL-1β signaling, inflammation, and fibrosis [45]. The mice with frameshift *NPPA* mutation elicited the most dramatic prolongation of QRS wave, slightly attenuated atrioventricular conduction and ventricular repolarization through the downregulation of the sodium channel in the atrium, ventricle, and atrioventricular junction [46]. In addition, ANP can reduce mRNA expression of Na_v_1.5 in the epithelium [47] and modulate *KCNQ1* expression [48]. Loss-of-function of Na_v_1.5 induced by its abnormalities of expression, trafficking, and location to the membrane, will lead to decreased sodium current, delayed activation, or earlier/faster inactivation, which can thus cause Brugada-like ECG or Brugada syndrome [49]. *NPPA* (p.R107X) and *NEBL* (p.R882X) mutations were identified in Brs patients. Our further analysis showed the indirect interaction between *NEBL* and *SCN5A* and the direct interaction between *NPPA* and *SCN5A.* Interestingly, there are high degrees of co-expressions among *NEBL*, *NPPA* and *SCN5A* in myocardial tissue. Therefore, we proposed that truncated mutations of *NPPA* (p.R107X) and *NEBL* (p.R882X) may induce Brugada syndrome by aberrantly affecting the cardiac sodium channel, similar to loss-of-function of the sodium channel.

### The common ionic-channel genetic mutations caused Brugada syndrome and Long QT syndrome

Our study also identified several pathogenic or likely pathogenic mutations of *SCN5A*, *KCNH2*, and *KCNQ1* in Brs and LQTs. The mutations of *SCN5A* (p.A1374S, p.R661W, and p.R965C) and *KCNH2* p.R692Q may be associated with Brs, which is consistent with previous studies [50–54]. *SCN*5A p.R965C can cause hyperpolarized inactivation and slower recovery from the inactivation of the sodium channel [55]. However, the mechanisms of how the mutations of *SCN5A* (p.A1374S and p.R661W) and *KCNH2* p.R692Q induce Brs are still unknown. Up to date, there is no functional research on the splicing mutation (c.922-1G > C) [56] and p.R243S [57–62] of *KCNQ1* demonstrated in LQTs. *KCNQ1* p.R243C can induce slower activation and the voltage dependence of activation and inactivation, which may shift to more positive potentials in the I_Ks_ channel. It can also impair the regulation by PKA and I_Ks_ channel-PIP_2_ (phosphatidylinositol 4, 5-bisphosphate) interactions. Therefore, it increases the risk of life-threatening events while having pronounced benefits from β-blocker treatment [57, 59, 60]. *SCN5A* p.R1880H (or p.R1898H), predicted to be a pathogenic mutation, has been previously reported in LQTs and Brs. It can dramatically reduce the sodium channel current [63, 64] and the abundance of Na_v_1.5 and N-Cadherin clusters at the intercalated disc, which is associated with ACM [65]. *KCNH2* p.D161N (similar to D501N) has been reported in cases of LQTs, even in a five-year-old boy of the ventricular non-compaction with LQTs [63, 66–69]. The *KCNH2* encodes 1159 amino acids of the α-subunit of voltage-dependent potassium channel mediator for the rapid component of delayed rectifying I_Kr_ current. For one LQTs case in our study, we also detected a novel and pathogenic frameshift mutation of *KCNH2* (p.A188Gfs*143). However, more than sixty patterns of frameshift mutations in *KCNH2* have been reported in LQTs [69]. For example, *KCNH2* p.G1006fs*49 can cause a significant delay in the voltage-sensitive transition to the channel open state, faster-inactivating kinetics, and quicker recovery from the inactivation for the delayed rectifying I_Kr_ current [70].

### *MYH7* and *MYH6* variants were identified in Brugada syndrome

According to a previous report, *DSG2* and *MYH7* have been identified as new potential Brs candidates [71]. The mutations of *MYH7* have been demonstrated in approximately 25% of patients with the overlap of hypertrophic cardiomyopathy and LQTs. Meanwhile, rare mutations of *MYH6* have also been identified in these patients [72]. In our study, *MYH7* (p.E1902Q) and *MYH6* (p.R1820Q) were predicted as "damaging/possibly damaging" and "damaging/damaging" by Polyphen and SIFT algorithms and were also identified in cases of Brs. However, whether these two variants cause Brs remains unclear, which needs further confirmation by more research center data and functional research.

## Limitations

The WES of blood DNA from these patients was completed before June 2017. This study was a retrospective study. We did not carry out the verification by Sanger sequencing for these mutations and variants. Our study needs further family genotype–phenotype co-segregation analysis and cell/animal research to investigate how the Brs and LQTs are associated with potential pathogenic mutations of *NEBL, NPPA, SCN5A*, *KCNH2* and *KCNQ1.*

## Conclusions

In our study, we first reported the indirect interaction between *NEBL* and *SCN5A* and the direct interaction between *NPPA* and *SCN5A*. There are high degrees of co-expressions among *NEBL*, *NPPA* and *SCN5A* in myocardial tissue. The truncated mutations of *NEBL* (p.R882X) and *NPPA* (p.R107X) may induce Brs by abnormally affecting the cardiac sodium channel. *SCN5A* (p.R661W, p.R965C and p.A1374S) and *KCNH2* (p.R692Q) may cause Brs, while *SCN5A* (p.R1880H), *KCNQ1* (c.922-1G > C and p.R243S) and *KCNH2* (p.D161N and p.A188Gfs*143) may lead to LQTs. Additionally, *MYH7* (p.E1902Q) and *MYH6* (p.R1820Q) were identified in Brs. However, further pedigree and functional research related to these mutations and variants are needed.

## Data Availability

The data used in this study is not publicly available, but it might be available from the corresponding author upon reasonable request and permission from relevant Chinese Authorities.

## Abbreviations

IPAS: Inherited primary arrhythmia syndromes
VT: Ventricular tachycardia
VF: Ventricular fibrillation
SCD: Sudden cardiac death
Brs: Brugada syndrome
LQTs: Long QT syndrome
WES: Whole exome Sequencing
ECGs: Electrocardiograms
ICD: Implantable cardioverter-defibrillators
SNPs: Single-nucleotide polymorphisms
ACM: Arrhythmogenic cardiomyopathy
PKP2: Plakophilin 2
DES: Desmin
DSP: Desmoplakin
NPPA: Natriuretic peptide precursor A
ANP: Atrial natriuretic peptide
AF: Atrial fibrillation
NEBL: Nebulette
KCNQ1: Potassium voltage-gated channel subfamily Q member 1
SCN5A: Sodium voltage-gated channel alpha subunit 5
MYH6: Myosin heavy chain 6
MYH7: Myosin heavy chain 7
KCNH2: Potassium voltage-gated channel subfamily H member 2
